# Conversion of fatty acid methyl esters into dibasic esters by metathesis and their lubricant properties†

**DOI:** 10.1039/d1ra04045f

**Published:** 2021-09-20

**Authors:** Jean-Luc Dubois, Jean-Luc Couturier, Svajus Joseph Asadauskas, Linas Labanauskas, Dalia Bražinskienė, Rolf Blaauw

**Affiliations:** Arkema, Corporate R&D 420 Rue d’Estienne d’Orves 92705 Colombes France; Centre de Recherches Rhône Alpes rue Henri Moissan - CS 42063 69491 Pierre Bénite France; Fiziniu ir Technologijos Mokslu Centras (FTMC) Sauletekio 3 Vilnius Lithuania asadauskas@chi.lt +370 5264 9360; Wageningen Food & Biobased Research, Wageningen University & Research Bornse Weilanden 9 Wageningen The Netherlands

## Abstract

Biodiesel plants are struggling to find value added applications for fatty acid methyl esters (FAME). One option for FAME valorization would be dibasic esters, which can be transesterified with 2-ethylhexyl (2EH) or other alcohols to produce lubricant basestocks and achieve the most widespread viscosity grades VG46 and VG32. Biocatalytic, metathesis and other synthetic pathways are available to produce dibasic esters. Using a ruthenium-based catalyst, methyl oleate was converted into monounsaturated dibasic ester by metathesis and reached VG22 after transesterification with 2EH in this investigation. Synthesized 2EH esters of other dibasic acids showed distinct viscometric trends. Their correlation implied that FAME from gondoic and erucic acids should result in VG32 and VG46 respectively, if converted into 2EH dibasic esters. Pour points demonstrated excellent low temperature fluidity and resistance to heat thinning when monounsaturation was retained. Oxidative stability properties remained acceptable, volatility was lower than that of VG46 mineral oils. Mixed alcohols, acids and esters can also be used for meeting VG specifications or achieving higher biobased contents. Currently petrochemical ester basestocks dominate in high performance hydraulic fluids (HF). However, fractionation of FAME into high-erucic/gondoic esters in biodiesel plants can produce a valuable biobased feedstock for large volume manufacture of HF and other lubricants.

## Abstract 2

Monounsaturated dibasic esters were obtained by FAME metathesis and tested for viscosity, extreme temperature and other lubricant properties. Their 2EH derivatives can produce 100% bio-derived basestocks for widespread heavy duty hydraulic fluids.

## Graphical abstract

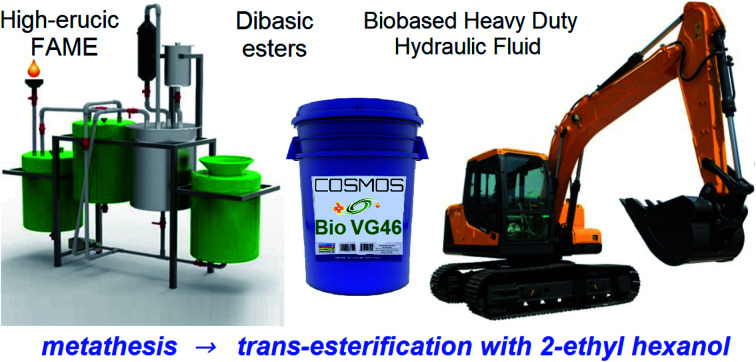

## 1. Introduction

With petroleum prices fluctuating around relatively low levels, biodiesel plants often have operational difficulties. Attempts are made to reduce raw material costs, streamline the manufacture and valorize the products, among which fatty acid methyl esters (FAME) are by far the most critical. As the feedstock, oils with unusual fatty acid (FA) profiles are sometimes used. While saturated FAME are often removed to improve low temperature fluidity, other FAME of various chain lengths or double bond positions typically remain intermixed with the dominating methyl oleate and linoleate. Only a few biodiesel plants consider the opportunity to fractionate FAME based on their chain lengths. Traditionally oleochemical industry focuses on free FA as a raw material for various niche applications. Segregation of individual free FA is quite complicated, because they are not easily fractionated and distillation often results in decarboxylation.^1^ Their FAME counterparts are much more subject to fractionation, because boiling ranges of FAME with C18, C20 or C22 FA are distinctly different. However, the demand for fractionated FAME is still limited, partly due to slow markets, such as the lubricant industry.

Among many lubricant raw materials, basestocks for hydraulic fluids (HF) represent an opportunity of high volume and substantial value for bio-derived chemicals. A broad selection of different esters is already utilized there, such as polyol esters, mellitates, estolides, soaps^2^*etc.* No dominant ester has emerged as a basestock yet, since these esters are still manufactured in batch processes. Many of them are difunctional, *e.g.* neopentyl glycol esters, dimer acid esters,^3^ phthalates, main constituents of estolides,^4^ glycol oleates, adipates, azelates, sebacates,^5^*etc.* The latter three belong to so called “dibasic esters”, which are colloquially defined as the esters of linear α–ω dicarboxylic acids. It might be noted that dibasic esters should be synthesized using C5 or preferably longer alcohol chains, such as 2-ethylhexyl (2EH). While in usage, shorter moieties tend to produce volatile carboxylic acids or ketones due to hydrolysis and oxidation of the esters. This is problematic in HF because of objectionable odors, corrosion issues and other side effects.

**Fig. 1:**
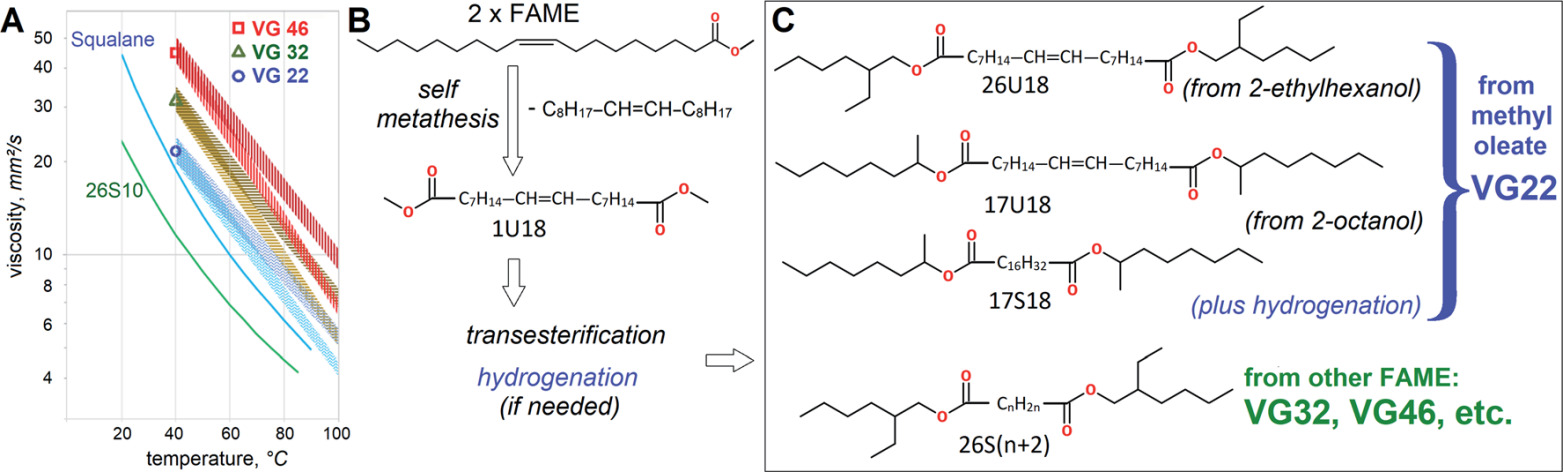
Effects of molecular architecture on Viscosity Grades (VG). Heat thinning trends with typical (VI = 100) and high (VI = 200) viscosity index. Reference data^21^ for squalane and di(2-ethylhexyl) sebacate (26S10) is provided for comparison (A). Scheme of self metathesis for C18:1 FAME (B). Transesterification of metathesis products with C_8_H_17_OH. Other alcohol moieties can also be used (C).

Despite broad availability of biobased esters, HF usually utilize petrochemical esters for better costs and performance. Viscosity grades VG46 and VG32 are by far the most dominating volume-wise, totaling over a million m^3^ worldwide.^6^ Formal VG specifications define HF viscosity at 40 °C, so VG46 should fall between 41.4 and 50.6 mm^2^ s^−1^, while VG32 between 28.8 and 35.2 mm^2^ s^−1^. Their heat thinning trends are also very important for heavy duty applications, because less heat-dependent viscosity assures better efficiency. In lubricant technology viscosity reduction with heating is defined by Viscosity Index (VI). Typically, mineral HF attain VI ∼100, ester-based HF thin down slower with VI ∼130 and vegetable oils stand out with VI ∼200 due to excellent resistance to heat thinning. Approximate variation of viscosity with temperature for VG22, VG32 and VG46 is shown in Fig. 1.

In essence, lower VG might be more favorable if equipment operates at higher temperatures, but uses HF of high VI. Consequently, despite lower volumes, VG32 is also very important, as manifested by wide usage of rapeseed oil in HF. Squalane and di-2EH-sebacate (26S10 in this report) are often used as control fluids in lubricant research^7^ and are employed to predict viscometric properties of dibasic esters theoretically.

The basestocks also need to address low temperature solidification and oxidative degradation, mostly related to acidification, vapor losses and residue formation. Cold properties can be improved by pour point depressants and oxidative stability by antioxidants, but they still mostly depend on the intrinsic basestock properties. A number of other technical aspects are also important for formulated HF. However, most of them are either dictated by basestock manufacture, such as clarity, color or contaminants, or can be resolved through the formulation with additives and system adaptation (lubricity, corrosion resistance, seal compatibility, water rejection, *etc.*). Additives for dibasic esters and vegetable oils are well established among HF formulators, so the latter properties rarely become extremely challenging.

Share of heavy duty biodegradable HF is growing and the main reason of slow penetration of biobased esters is the absence of basestocks, which would be manufactured in a continuous process. Since the widespread presence of biodiesel plants provides assured supply of FAME, including those from industrial crops,^8^ the concept of using FAME for the basestock manufacture has already been pursued.^9,10^ However, VG32 or VG46 is still challenging to achieve by using just dibasic esters and polyol esters, which have already been well established as mainstream lubricant basestocks. Conversion of FAME into dibasic esters of VG32 or VG46 would have a better industrial appeal than an introduction of a more unusual molecular architecture from less abundant feedstocks. The industrial potential of dibasic esters is further enhanced by rapid development of metathesis processes, which is a promising synthetic mechanism for oleochemicals. Metathesis capitalizes on the presence of double bonds to restructure hydrocarbon chains or heterocycles.^11^ In most cases, this process involves so called ‘self-metathesis’, which is nearly impossible to avoid, because unsaturated molecules can react between themselves. In case of FAME, *e.g.* methyl oleate, self-metathesis would lead to a monounsaturated difunctional methyl ester of α,ω-C18:1-Δ9 dibasic acid.^12^ Self-metathesis of FAME has already been attempted for several applications, but the HF basestock has only been pursued in patent literature.^13^ Olefins from FA metathesis had already been considered for motor oil basestocks.^14^ Therefore, the side products of self-metathesis can also be utilized for lubricants, just not as ester, but as hydrocarbon basestocks.

In this investigation a series of conventional 2EH dibasic esters (adipate, sebacate, *etc.*) are compared to innovative dibasic esters with or without monounsaturation as products of FAME metathesis and transesterification with 2EH or other alcohols. The focus is devoted to FAME of oleic, gondoic and erucic acids, the former being a predominant component of biodiesel plant products. The latter FAME are present in various quantities depending on the origins of transesterified oils. Erucic acid may even be the most abundant FA in some rapeseed varieties, crambe and other non-food oils. Synthesis and viscometric testing provides sufficient data to identify which FAME would be the most suitable for conversion into VG32 and VG46 basestocks. Many properties have to be attained by fully formulated HF, such as lubricity or corrosion inhibition, but they can mostly be controlled by additives and manufacture practices. Therefore, in this investigation the experiments are limited to low temperature fluidity, volatility and oxidative stability testing, *i.e.* the properties which are the most relevant to the basestock itself.

## 2. Experimental

Original descriptions of materials and methods have already been provided in patent literature.^13,15^ In this section, they are summarized only briefly. Most investigated dibasic esters are itemized there also along with their spectral data. Two newly synthesized dibasic esters and new methods are described below in detail.

## 3. Materials

A series of dibasic esters are studied along with selected oils for comparison. Most dibasic esters have been pre-synthesized in-house as described previously,^13,16^ although several of them have been acquired from external suppliers. Their codes, names and sources are provided in Table 1.

**Table 1:** List of dibasic esters, tested in this study along with their compositional moieties, names and sources or synthesis description references

Code	Alcohol moiety	Acid moiety	IUPAC, colloquial or trade name	Ref or supplier
1U18	Methanol	α–ω C18:1	1,18-Dimethyl octadec-9-enedioate	16
17S18	2-Octanol	α–ω C18:0	1,18-Di(1-methylheptyl) octadecanedioate	13
17U18	2-Octanol	α–ω C18:1	1,18-Di(1-methylheptyl) octadec-9-enedioate	13
26S18	2-Ethylhexanol	α–ω C18:0	1,18-Di(2-ethylhexyl) octadecanedioate	13
26U18	2-Ethylhexanol	α–ω C18:1	1,18-Di(2-ethylhexyl) octadec-9-enedioate	19
37U18	2-Propylheptanol	α–ω C18:1	1,18-Di(2-propylheptyl) octadec-9-enedioate	13
48U18	2-Butyloctanol	α–ω C18:1	1,18-Di(2-butyloctyl) octadec-9-enedioate	13
26S9	2-Ethylhexanol	α–ω C9:0	Di(2-ethylhexyl) azelate	TCI
26S10	2-Ethylhexanol	α–ω C10:0	Di(2-ethylhexyl) sebacate	TCI
26S12	2-Ethylhexanol	α–ω C12:0	1,12-Di(2-ethylhexyl) dodecanedioate	TCI
26S16	2-Ethylhexanol	α–ω C16:0	Di(2-ethylhexyl) thapsate	New synthesis
26S20	2-Ethylhexanol	α–ω C20:0	1,22-Di(2-ethylhexyl) eicosanedioate	New synthesis

The codes reflect the chain lengths of the branches in alcohol and the dibasic acid moieties of the specific molecule. The alcohol moiety segments are encoded in the prefix depending on the number of C atoms in the branches of the alcohol. For example, 2-octanol contains branches of C1 and C7, while 2EH has those of C2 and C6. Hence the prefixes of “17” and “26” are assigned respectively. The central character of the code represents whether the dibasic ester is fully saturated (“S”) or mono-unsaturated (“U”). The tail of the code denotes the number of C atoms in the dibasic acid. For example, thapsic acid (α–ω C16:0) and α–ω C18:1 dicarboxylic acid are assigned “16” and “18” respectively. Sometimes several dibasic esters are bundled into one group, such as “26Sxx” (2EH esters of saturated dibasic acids), “xxS18” (octadecanedioates) or “xxU18” (octadecenedioates).

In addition to dibasic esters, several oils are studied for comparison, representing reference compounds and commercial basestocks, Table 2. These can be subdivided into two groups: (1) Mineral Basestocks “MinB” and (2) Synthetic Basestocks “SynB”. Technically, it is not quite correct to delegate the biobased low erucic acid rapeseed oil (LEAR) into the group of synthetic materials. However, this report might become too confusing if LEAR were given a separate distinction.

**Table 2:** List of oils, used for comparison in this study along with affiliation to mineral basestocks (“MinB”) and synthetic basestocks (“SynB”)

Code	Type	Group	Description/tradename	Manufacturer
RL208	Mineral oil	MinB	NOACK reference oil RL208	Shell
350N	Mineral basestock	MinB	Paraffinic mineral oil, HVI 350™	Shell
LEAR	Vegetable basestock	SynB	Low erucic rapeseed oil, food grade	Maxima
PAO8	Synthetic basestock	SynB	Poly-α-olefin, Durasyn 168™	Ineos
Squalane	Pure compound	SynB	2,6,10,15,19,23-Hexamethyltetracosane	TCI

Oils 26S9, 26S10 and 26S12, representing fully saturated α–ω dibasic esters (azelate, sebacate and dodecanedioate respectively), squalane, thapsic (1,16-hexadecanedioic acid) and 1,20-eicosanedioic acids were purchased from TCI Chemicals (Belgium). Low Erucic Acid Rapeseed oil (LEAR), distributed by the grocery chain Maxima (Lithuania) was purchased in a local store. Polyalphaolefin PAO8 was received from Ineos Oligomers (USA) as Durasyn 168. Mineral oil RL208 was manufactured by Shell and purchased from Weber Reference Oils (Germany). Reagent grade 2-octanol (97%), 2-ethylhexanol (2EH alcohol, 99%), 2-butyloctanol (95%), 4 *N*,*N*-dimethylaminopyridine (DMAP, 99%), dichloromethane (DCM, 99.5%) and dicyclohexyl carbodiimide (DCC, 99%) were purchased from Sigma-Aldrich. Perstorp Inc. (Sweden) kindly provided a free sample of 2-propylheptanol.

Some reference oils contained various compounds of different mol. wt Synthetic and vegetable oils usually have an identifiable component, which is much more abundant than others. Glycerol trioleate (892 g mol^−1^) was selected to represent mol. wt of LEAR, since LEAR is mostly composed of oleate glycerides (over 60 mol%). Several researchers investigated PAO8 composition chromatographically, concluding that C_40_H_82_ (563 g mol^−1^) should be the most abundant hydrocarbon.^17^ In mineral oils it would not be reasonable to identify any particular molecule to represent the whole mixture. So a typical practice to calculate the average mol. wt of mineral basestocks using ASTM D2502 was employed, relying on their viscosities at 100 °F (*i.e.* 37.8 °C) and 210 °F (*i.e.* 98.9 °C). The improved approach which also uses density values^18^ was employed:mol. wt = 223.56·*v*_100F_^(1.1228·*d* − 1.2435)^·*v*_210F_^(3.038·*d* + 3.4758)^·*d*^−0.6665^where *d* is density (g mL^−1^) at 15.6 °C; *v*_100F_ and *v*_210F_ are kinematic viscosities (mm^2^ s^−1^) at 37.78 °C and 98.89 °C respectively.

### Synthesis

The dibasic esters were mostly synthesized from methyl oleate by following the scheme in Fig. 1. Self-metathesis of methyl oleate took place concurrently with cross-metathesis process as described previously^16^ with the use of ruthenium–carbene catalysts. The products were separated by distillation to segregate dimethyl esters 1U18 from remaining FAME, C_18_H_36_ olefin and other metathesis compounds. Then 1U18 was transesterified with 2-octanol, 2EH, 2-propylheptanol or 2-butyloctanol to yield respective monounsaturated dibasic esters with 95%+ purity. Transesterification was carried out as described previously^13^ using *p*-toluene sulfonic acid as a catalyst and 5 mol% excess of the respective alcohol. Several of them were hydrogenated to eliminate the double bonds using palladium/carbon catalyst in ethyl acetate. In order to synthesize thapsate and eicosanedioate dibasic esters, direct esterification was performed using 4 *N*,*N*-dimethylamino pyridine (10 mol% excess) in dichloromethane with dicyclohexyl carbodiimide.

Syntheses of 2EH esters of thapsic acid (26S16) and eicosandioic acid (26S20) have not yet been reported. Thapsate was synthesized in a solution of thapsic acid (5 g; 17.4 mmol) and DCM (500 mL) by adding 2EH alcohol (4.8 g; 37 mmol), DMAP (0.21 g; 1.74 mmol) and DCC (7.42 g; 36 mmol). Reaction mixture was stirred for 72 hours at 35 °C. Precipitated solid was filtered off, DCM was removed under reduced pressure. Residue was dissolved in hexane (200 mL) and solution was eluted (ethyl acetate and hexane mixture 1 : 29, vol.) through a pad of silica gel (5 cm height). Solvent was evaporated on rotary evaporator, and residual volatiles were removed under reduced pressure (0–1 mm Hg). Obtained di(2-ethylhexyl) hexadecanedioate (code 26S16) appeared as light amber liquid (6.8 g, 77% yield). ^1^H NMR (400 MHz, CDCl_3_): *δ* 3.98–3.84 (m, 4H, –O–CH_2_–), 2.23 (t, *J* = 7.5 Hz, 4H, C=OCH_2_–), 1.60–1.45 (m, 6H, –CH; –C=OCH_2_–CH̲_2_–), 1.34–1.08 (m, 36H, –CH_2_–), 0.82 (t, *J* = 7.4 Hz, 6H, –CH_3_), 0.81 (t, *J* = 7.4 Hz, 6H, –CH_3_).^13^C NMR (100 MHz, CDCl_3_): *δ* 174.09 (C=O), 66.61 (–O–CH_2_), 38.77 (CH), 34.45 (C=OC̲H_2_–), 30.43, 29.63 29.60, 29.48, 29.28, 29.17, 28.92, 25.06, 23.81, 22.97 (–CH_2_–), 14.03 (–CH_3_), 10.99 (–CH_3_).

Synthesis of 2EH ester of eicosandioic acid (26S20) was carried out in a solution of 1,20-eicosandioic acid (10.3 g; 30 mmol) and DCM (500 mL) by adding 2EH alcohol (9.11 g; 70 mmol), DMAP (0.85 g; 7 mmol) and DCC (14.4 g; 70 mmol). Reaction mixture was stirred for 72 hours at 35 °C. Precipitated solid was filtered off, DCM was removed under reduced pressure. Residue was dissolved in hexane (200 mL) and solution was eluted (ethyl acetate and hexane mixture 1 : 19 vol.) through a pad of silica gel (5 cm height). Solvent was evaporated on rotary evaporator, and residual volatiles were removed under reduced pressure (0–1 mmHg). Obtained di(2-ethylhexyl) eicosanedioate (code 26S20) appeared as light amber liquid (11.4 g, 75% yield). ^1^H NMR (400 MHz, CDCl_3_): *δ* 4.05–3.94 (m, 4H, –O–CH_2_–), 2.31 (t, *J* = 7.5 Hz, 4H, C=OCH_2_–), 1.69–1.50 (m, 6H, CH, –O–CH_2_–CH̲_2_–), 1.41–1.23 (m, 44H, –CH_2_–), 0.91 (t, *J* = 7.4 Hz, 6H, –CH_3_), 0.90 (t, *J* = 7.4 Hz, 6H, –CH_3_).^13^C NMR (100 MHz, CDCl_3_): *δ* 174.09 (C=O), 66.61 (–O–CH_2_–), 38.77 (CH), 34.46 (C=OC̲H_2_–), 30.44, 29.69, 29.68, 29.65, 29.61, 29.48, 29.28, 29.17, 28.93, 25.06, 23.81 and 22.97 (–CH_2_–), 14.03 (–CH_3_), 10.99 (–CH_3_).

**Fig. 2:**
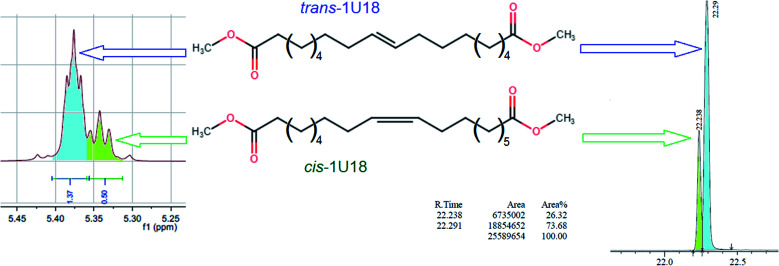
Distribution of *trans* and *cis* isomers in the dibasic acid moiety in NMR spectra and GC chromatograms, as adapted.^13^

It should be noted that due to metathesis-based synthesis monounsaturated dibasic esters represent a mixture of *cis*- and *trans*-isomers, so the obtained xxU18 esters cannot be considered as individual organic compounds. Distribution between the *cis*- and *trans*-isomers in 1U18, which was used for synthesizing xxU18 was analyzed by NMR and GC as described,^16^ see Fig. 2. It was established by the two techniques that the molar ratio of *trans*-/*cis*-isomers was 73.3/26.7 and 73.7/26.3 respectively, showing an excellent agreement. Roughly, *trans*-isomers are nearly three times more abundant than *cis*-isomers.

### Fluidity measurements

The procedures include measurements of viscosities and low temperature fluidity, *i.e.* pour point. Kinematic viscosities were measured by adapted ASTM D445 procedure using Cannon-Fenske capillary viscometers, maintaining 40 °C or 100 °C temperatures with ±0.1 °C accuracy. In this report the term “viscosity” refers exclusively to kinematic viscosity, unless specified otherwise. Viscosity values from literature sources were also used for discussion. When dynamic viscosities were extracted, they were divided by density and converted into kinematic viscosities. When viscosities at 100 °F and 210 °F were extracted, they were converted into kinematic viscosities at 40 °C and 100 °C respectively using ASTM D341 protocol. Viscosity Index (VI) was calculated using ASTM D2270 methodology. Pour points were measured using an adapted ASTM D97 procedure for low-volume samples.^19^ As required by the standard, sample pouring was inspected at 3 °C increments using +21 °C as a starting temperature. Each sample was tested at least twice, but no attempt was made to make runs at other starting temperatures. As implied by the ASTM D97 standard, the measurement procedure of pour points below −57 °C was not extended to lower temperatures.

### High temperature testing

The procedures include measurements of volatility using thermogravimetric analysis (TGA) and oxidative stability using thin film oxidation tests. NOACK volatility protocol of ASTM D6375 was employed by using Pyris TGA1 instrument (PerkinElmer). Initially, 9–10 mg oil samples were placed in a platinum crucible of 5.5 mm ID and a 2.8 mm height. Reference oil RL208 was used for calibration to establish the air flow and heat ramping parameters. The flowrate of 20 mL min^−1^ was applied and initially the thermal ramping of 50 °C min^−1^ was used for the temperature range of 50–235 °C. Afterwards, the ramping was reduced to 5 °C min^−1^ until reaching 249 °C and then the sample was held for 4 minutes under isothermal conditions. Oxidative degradation was screened using a thin film oxidation procedure as described in detail previously.^20,21^ Vapor losses were measured gravimetrically by placing the oil sample in a film of 500 μm thickness on a steel coupon and heating it at 120 °C under ambient humidity. The coupons were weighed periodically to monitor the vaporization rate. Polymerization also took place during the tests and some samples formed insoluble residues. The duration until more than half of the residual film volume was no longer soluble in acetone was recorded. Two or more runs were carried out for each oil both in NOACK volatility and thin film oxidation tests.

## 4. Results and discussion

Out of many lubricant properties, viscosity is often the most important, in particular for HF. Therefore, this report devotes most attention to the relationships between molecular architecture of dibasic esters and their viscosities. One of the major goals is to establish which particular FAME is suitable for conversion into VG32 or VG46 basestocks. These viscosity grades are by far the most popular in HF.

Many other properties are also important for the basestocks, such as low-temperature fluidity, volatility, oxidative stability, heat capacity, ability to dissolve additives and perform well in lubricity, corrosion and other bench tests. Good performance in many of these tests have already been demonstrated in previous reports^6,13^ for the most promising dibasic esters. Their conclusions are summarized in the below subchapters along with the results of the oxidative stability experiments.

### Comparison of viscometric properties

Synthesized dibasic esters, including those described previously^13^ or acquired from suppliers, show a rather broad range of properties. Viscosities of some these dibasic esters were also reported by other researchers.^22^ Their data^23^ along with the results of this study, including volatility and pour point measurements, are itemized in Table 3. It can be observed that some viscosities and especially VI appear noticeably different among the reports. Viscosity measurement accuracy might be related to the compound purity, equilibration of correct temperature or other experimental issues. Pour point and VI values are cited in Table 3 as reported in the references, so it remains uncertain how the latter were calculated. Pour points below −60 °C cannot be measured in strict compliance with ASTM D97, so their values from the references should be viewed with some caution. Only those values that were measured and calculated in this study are used for the below artwork. In addition to the dibasic esters, several other basestocks and reference oils were employed for comparison. They are itemized in the same manner, Table 3.

**Table 3:** Measured viscometric, low temperature fluidity and NOACK volatility properties of α–ω dibasic esters and comparative oils along with some reference values

Sample code	mol. wt, g mol^−1^	Viscosity at 40 °C, mm^2^ s^−1^	Viscosity at 100 °C, mm^2^ s^−1^	VI	Pour pt, °C	NOACK, wt%	Oil type	Reference
1U18	340.49	8.68	2.85	204	+21	28.4%	xxU18	13
17S18	538.88	22.7	5.31	180	+9	2.7%	xxS18	13
17U18	536.88	21.2	5.35	205	−21	2.0%	xxU18	13
26S18	538.88	23.94	5.71	194	−9		xxS18	13
26U18	536.88	22.74	5.53	197	−57	3.7%	xxU18	13
37U18	592.97	28.74	6.39	174	−51	2.0%	xxU18	13
48U18	649.1	36.09	7.09	163	−54	2.5%	xxU18	13
26S6	370.57	7.71	2.22	90	<−60	30.4%	26Sxx	This report
		7.73^a^	2.34^a^	121	<−67			22
		8.43	2.35	90	−76			5
26S9	412.64	10.52	2.8	111	<−60	15.0%	26Sxx	This report
		10.31^a^	2.93^a^	138	<−62			22
26S10	426.7	11.57	3.28	164	<−60	9.4%	26Sxx	This report
		11.59^a^	3.26^a^	160				23
		11.52	3.07	121	−65			5
		11.97^a^	3.28^a^	147	<−62			22
26S12	454.72	14.72	3.72	147	−48	5.3%	26Sxx	13
26S16	510.83	17.9	4.1	134	−18	4.6%	26Sxx	This report
26S20	594.9	29	6.5	189	−3	3.9%	26Sxx	This report
LEAR	892	34.72	8.29	229	−24	1.7%	SynB	This report
PAO8	563	50.48	8.2	135	<−60	1.8%	SynB	This report
350N	415	61.4	8.46	109	−15	7.2%	MinB	This report
RL208	490	38.56	6.8	102	−18	12.4%	MinB	This report
Squalane	422.81	18.78	4.05	115	<−60	11.2%	SynB	This report
		18.84^a^	4.08^a^					23
		20.9	4.2	103				24

a Yielded by conversion from mPa s and correlation using ASTM D341.

It should be noted that it was necessary to convert some viscosities, extracted from literature, or assume average mol. wt for some oils using ASTM D341 or D2502 as described above. For verification, the conversion yields 550 and 395 g mol^−1^ for PAO8 and squalane respectively. Compared to 563 and 422.81 g mol^−1^ in Table 3, these values are 2.3% and 6.5% lower. In contrast to mineral oils, PAO8 and squalane are free of aromatics and heterocycles, which might affect viscosity significantly. Therefore, the adapted ASTM D2502 values are employed only for RL208 and 350N. Calculated mol. wt values appear useful for correlating viscometric and evaporation properties and comparing dibasic esters to other basestocks, used for HF or at least to the established reference compounds. In this study, several well-recognized oils are employed as controls. Despite their relatively low mol. wt, 26S9 and 26S10 (azelate and sebacate) are occasionally used as synthetic basestocks for VG12. LEAR is frequently used as a basestock for vegetable oil-based HF. PAO8 is a widespread synthetic basestock for many lubricants. Mineral oil RL208 is used as a calibration fluid for various lubricant tests, including NOACK volatility. Squalane is often employed as a simulation compound^7^ to represent basestocks. Their viscometric, extreme temperature and degradation trends are compared to those of dibasic esters in the sections below.

### Correlation and prediction of viscosity grades

**Fig. 3:**
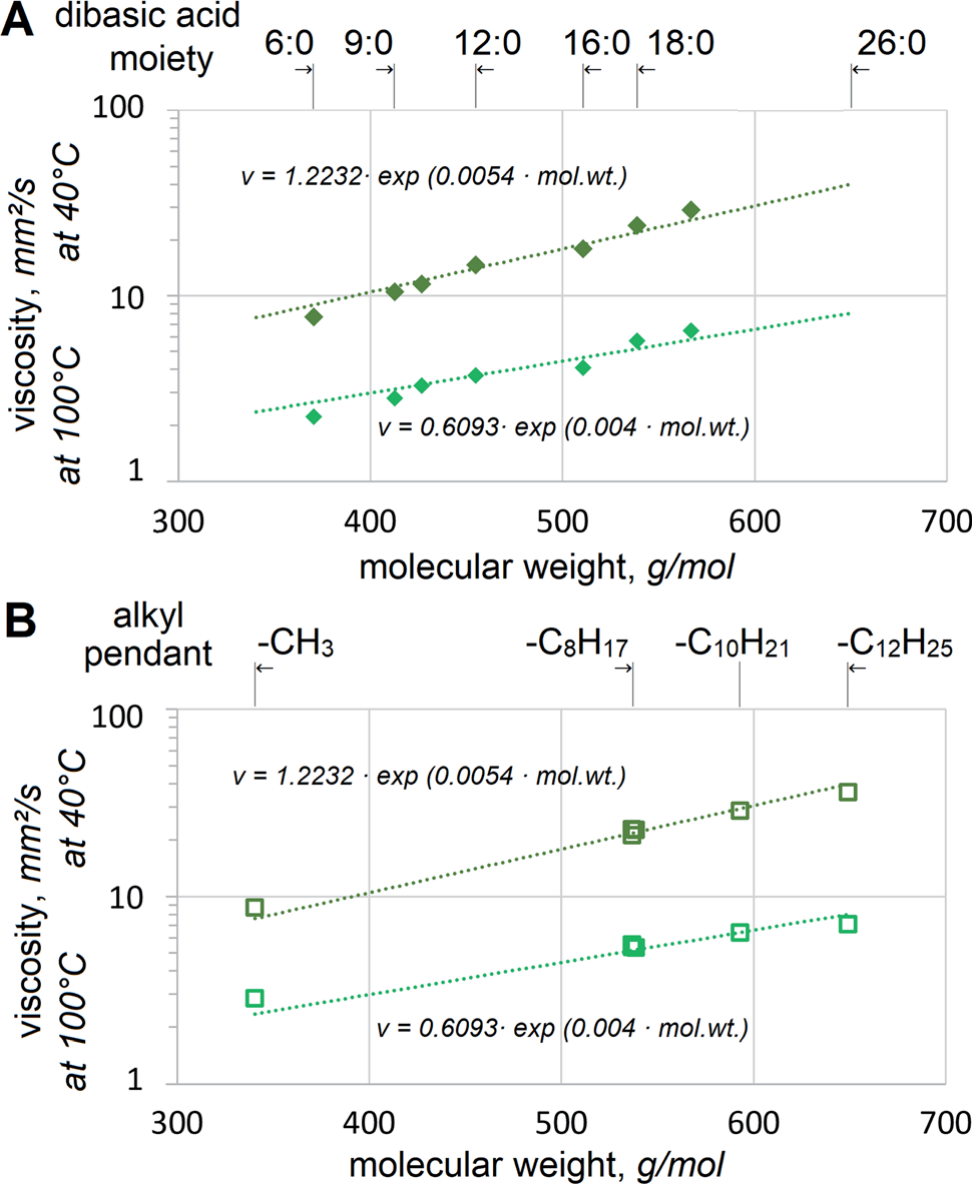
Influence of mol. wt on viscosity at 40 °C (darker symbols) and 100 °C (lighter symbols) of (A) 2EH esters of saturated dibasic acids and (B) the esters of α,ω-18:0 or α,ω-18:1 dibasic acids. Trendlines reflect the combined data from A and B.

Adipate, azelate and sebacate dibasic esters have been used in lubricants for many decades. However, their viscosity is too low to be utilized as basestocks for VG32 and VG46 hydraulic fluids. Since 2EH alcohol is the most popular and usually the most cost-effective choice in dibasic esters, 2EH esters with saturated dibasic acids are given higher priority industrially. Its biobased version is also available on the market. Viscosities of 2EH esters of various dibasic acids clearly depend on their mol. wt, Fig. 3(A).

The relationship between viscosity and mol. wt appears to approach exponential both at 40 °C and 100 °C. This agrees well with the expectations, as reported by other researchers for fatty esters^25^ or hydrocarbons.^26^Fig. 3 shows the aggregate trendlines for all dibasic esters, established as *v* = 1.2232·exp(0.0054·mol. wt) with *R*^2^ = 0.9704 at 40 °C and *v* = 0.6093 exp(0.004 mol. wt) with *R*^2^ = 0.916 at 100 °C. If viscosities at 40 °C per VG specifications are considered, the trendlines imply that 32 mm^2^ s^−1^ would be reached at 604.5 g mol^−1^ and 46 mm^2^ s^−1^ at 671.7 g mol^−1^. These values approach those of possibly industrially viable self-metathesis products, α,ω-C22:1 from FAME with Δ11 (*e.g.* methyl gondoate C20:1-Δ11) and as α,ω-26:1 from FAME with Δ13 (*e.g.* methyl erucate). Considering the former, a similar value of 32.85 mm^2^ s^−1^ at 40 °C for 2EH-terminated mixed diesters of monounsaturated α,ω-dicarboxylic acids HOOC–C_20_H_38_–COOH has already been reported.^27^ Despite the same mol. wt those acids contain methyl branching, which might affect viscosity somewhat, but the difference is not likely to be large. This confirms that the esters of 2EH and dicarboxylic α,ω-C22 or α,ω-C26 acids with or without methyl branches should have excellent viscosities for VG32 or VG46. It should be noted that if biobased 2EH is used, its esters with α,ω-C22 or α,ω-C26 would constitute 100% bio-derived VG32 or VG46 respectively.

As a matter of fact, formulated HF contain additives, such as antioxidants, anti-wear agents, rust inhibitors and many others, including several wt% of relatively high mol. wt polymers. Addition of 1–2% of viscosity index improvers^28^ and pour point depressants^29^ should lead to a viscosity increase by 10–20%. The thickening effect would comfortably accommodate the formulation into the needed VG. Therefore, gondoate- and erucate-based FAME might provide a valuable feedstock for the manufacture of high volumes of HF. Among industrial crops, gondoic acid is present at significant levels in *Simmondsia chinensis* (jojoba), *Camelina sativa* (camelina) and several other oilseeds. Availability of erucic acid is much broader, including high-erucic rapeseed, wallflower, mustard, *Crambe abyssinica* (crambe) and other sources. If these oils are processed in biodiesel plants, distillation of FAME can be a viable option for obtaining methyl erucate feedstocks of sufficient purity for metathesis as well. It should be noted that biocatalytic pathways to selectively hydrolyze and transesterify erucic a.^30^ or convert erucic a. in to α,ω-C22:1 have already been reported,^31^ which provides one more incentive to fractionate FAME and produce dibasic esters for HF.

Another option to achieve VG32 or VG46 viscosities might be esterification with other alcohols, heavier than 2EH. Esters of several Guerbet alcohols with saturated and monounsaturated C18 dibasic acids are compared, Fig. 3(B). The dibasic esters also include those with methyl and 2-octanol moieties. Again, good correlation with mol. wt can be observed. Monounsaturation appears to bring viscosity down, albeit only very slightly, when comparing 17S18 *vs.* 17U18 or 26S18 *vs.* 26U18. This agrees with reports on viscosities of stearate, oleate and linoleate esters,^25^ which find lower viscosity with increasing unsaturation. If 17S18 *vs.* 26S18 or 17U18 *vs.* 26U18 are compared, no significant dependence of viscosity on either methyl or ethyl branches could be observed. It seems that alcohols heavier than 2-butyl octanol would be beneficial to approach VG46. However, it is very problematic to use heavy alcohols for industrial (*trans*-)esterification. Even under high alcohol excess, the reaction typically does not go to completion, with significant levels of partial esters and free alcohols still present. These need to be distilled off and it becomes necessary to employ deep vacuum and high temperatures. The high temperatures needed for distilling alcohols heavier than ∼C_13_H_27_OH lead to the side reactions of progressively larger scale. In addition, high costs of such alcohols and the lack of biobased options present even further limitations. Therefore, the approach of using heavier alcohols and high-oleic FAME appears less viable than the one above, based on 2EH and high-erucic FAME, see Fig. 1C.

**Fig. 4:**
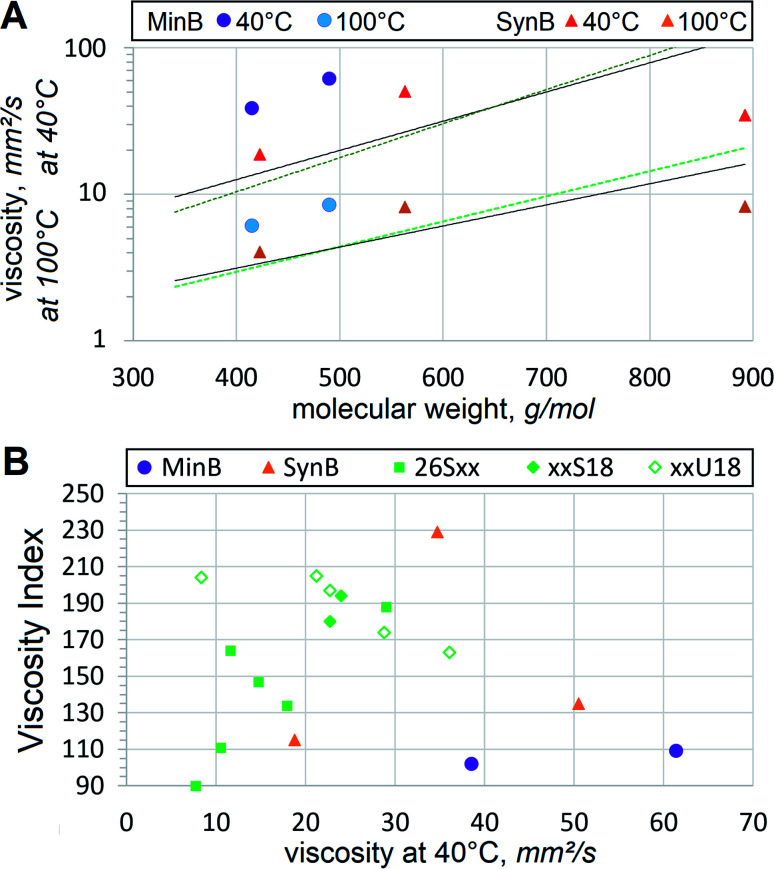
Viscometric trends showing (A) influence of mol. wt on viscosity at 40 °C (darker symbols) and 100 °C (lighter symbols) of mineral (MinB) and synthetic (SynB) basestocks. Continuous trendlines show the aggregate viscosity *vs.* mol. wt correlation of C22:1 α,ω-diesters,^27^ dotted trendlines from Fig. 3 are utilized; (B) differences in VI between several types of oils.

It should also be noted that viscosities of dibasic esters appear significantly lower than those of hydrocarbons of similar mol. wt, Fig. 4(A). Lower-than-expected viscosity of dibasic esters presents another advantage, because thinner oil is often beneficial, while volatile emissions are usually detrimental to lubricant basestocks. Compared to dibasic esters, hydrocarbons of similar viscosity would have lower flash points, faster evaporation and other volatility-related problems. Suitability of dibasic esters for high temperature applications is even more evident when VI is compared, Fig. 4(B).

Oil refiners continuously strive to achieve higher VI for mineral basestocks. Conventional crudes commonly have VI below 100, therefore, hydroisomerized and other deeply refined basestocks have been developed. Nevertheless, they rarely exceed VI of 140, even PAO8 has VI = 135. Dibasic esters demonstrate clearly higher VI than those of mineral oils or synthetic hydrocarbons. In most cases heat thinning of dibasic esters is slower than that of polyol esters or other niche basestocks. Vegetable oils, such as LEAR with VI = 229, represent the only basestock type that can offer higher VI, but that comes at the expense of oxidative stability and low temperature fluidity. Molecular linearity and abundance of unsaturation, in particular *cis*-unsaturation (which would be much higher if dibasic acids are produced enzymatically from FAME), are the main factors that contribute to VI. Monounsaturation seems to benefit VI in dibasic esters as well, when comparing 17S18 *vs.* 17U18 or 26S18 *vs.* 26U18. Slow heat thinning is very important for HF, leading to better energy savings, less wear and improved longevity of hydraulic systems. This tendency of dibasic esters, fortified by lower-than-expected volatility and viscometric suitability for VG32 or VG46, are very helpful in utilizing those basestocks for heavy duty HF.

### Influence of operating temperatures

Viscometric parameters are usually most important for the lubricant basestocks, but a series of other properties must also be considered. In case of HF, the range of operating temperatures must also be assessed. Hydraulic equipment might need to start up in cold winter and *vice versa*, it has to operate for long hours in sunny hot conditions. Under those circumstances volatility and oxidation may become important, as discussed below. With respect to another thermal extreme, HF should resist crystallization and solidification at low temperatures.

**Fig. 5:**
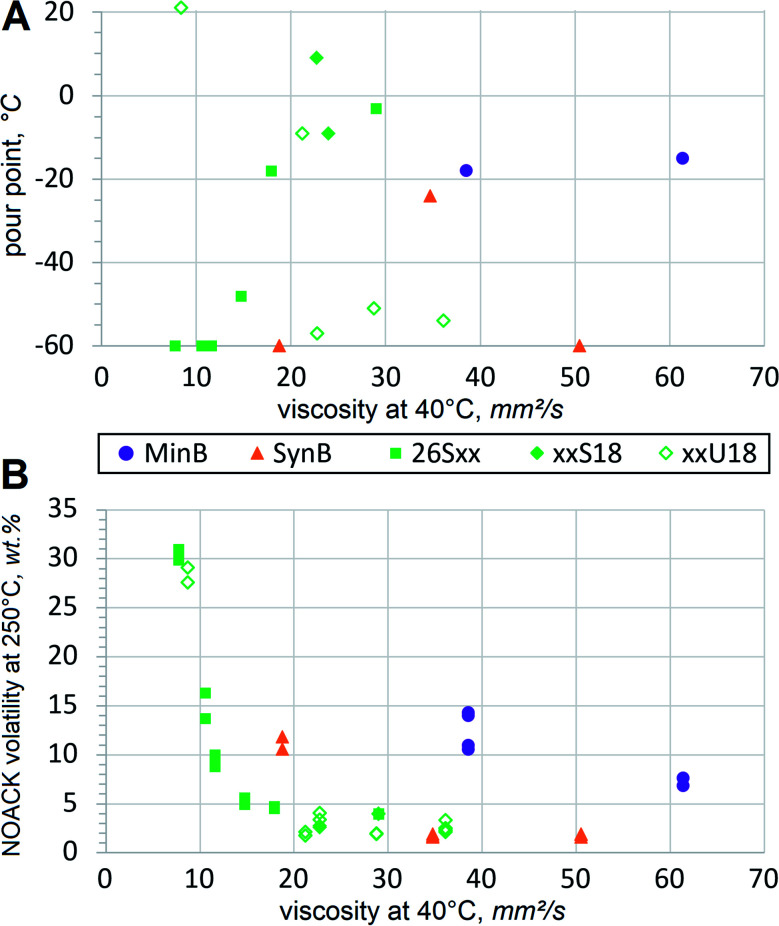
Phase transitions at thermal extremes of dibasic esters and conventional HF basestocks: (A) low temperature solidification as pour points and (B) evaporation at 250 °C as all recorded NOACK volatility values. Actual pour points, displayed at −60 °C, might be lower due to detection limits.

In order to assess low temperature fluidity, pour points are typically measured. A pour point can be perceived as an opposite of the melting point, *i.e.* it defines the temperature at which the liquid stops pouring when cooled down gradually. Pour points usually go up with increasing mol. wt, but branching, double bonds and other structural features also have strong influence. Some trends become evident when comparing pour points of dibasic esters with those of conventional basestocks, Fig. 5(A).

Pour point dependence on mol. wt is evident when comparing the 26Sxx homologues. Exact values are also listed in the Table 3 to relate them to mol. wt. Adipate, azelate and sebacate (26S6, 26S9 and 26S10) solidify at lower temperature than dodecanedioate (26S12), thapsate (26S16), 26S18 and 26S20. Unsaturated xxU18 dibasic esters do not show much correlation, first of all due to the high pour point of the methyl-terminated dibasic ester 1U18, which can be considered a linear molecule. Methyl-branched 17U18 also shows relatively high melting. But when the branching alkyls are longer, as in ethyl, propyl and butyl (26U18, 37U18 and 48U18 respectively), pour points drop down significantly, approaching that of PAO8. More sophisticated techniques would be necessary to evaluate fluidity below −60 °C. Such extremely low temperatures might be important in niche applications, such as arctic or aerospace, but for conventional HF fluidity below −40 °C is considered fully sufficient. Pour points of monounsaturated dibasic esters are much lower than those of mineral basestocks or LEAR, and such performance at low temperature constitutes a major advantage. Excellent low temperature properties of dibasic ester formulations would be far superior to those of conventional HF, especially when operating in colder climate.

At another thermal extreme of high temperatures, volatile losses are not desirable in HF. So the presence of low mol. wt components should be avoided, because volatility cannot be significantly reduced by using some additives. A standard TGA test for the basestock volatility is performed using a NOACK protocol at 250 °C. Mineral oil RL208 shows by far the fastest evaporation at 11.7% despite its viscosity being above VG32 or most of the tested esters. Since mineral oils contain broad diversity of hydrocarbons and heterocycles with varying mol. wt, it can be expected that some constituents are more volatile and evaporate more rapidly. Synthetic basestock PAO was specified at 2.8% NOACK volatility^32^ and recorded 1.8%. Evaporation of xxS18 and xxU18 dibasic esters appears comparable, Fig. 5(B). As expected, 26Sxx dibasic esters of lower mol. wt record higher numbers. Volatile contaminants have more influence on the test results in higher mol. wt materials, so this procedure is not very suitable to differentiate between low volatility basestocks, such as PAO or LEAR. For example, conventionally refined and deodorized food grade vegetable oils usually produce NOACK values below 2%.^33^ Nevertheless, the acquired test results confirm that emissions from FAME-derived dibasic esters are much lower than those from mineral basestocks. The dibasic esters should be considered similar to the recognized low volatility basestocks, such as PAO8 or LEAR. Formulations with these basestocks demonstrate increased flash points, absence of objectionable odors and improved resistance to fire hazards, all of which are very beneficial for HF.

### Resistance to oxidation

**Fig. 6:**
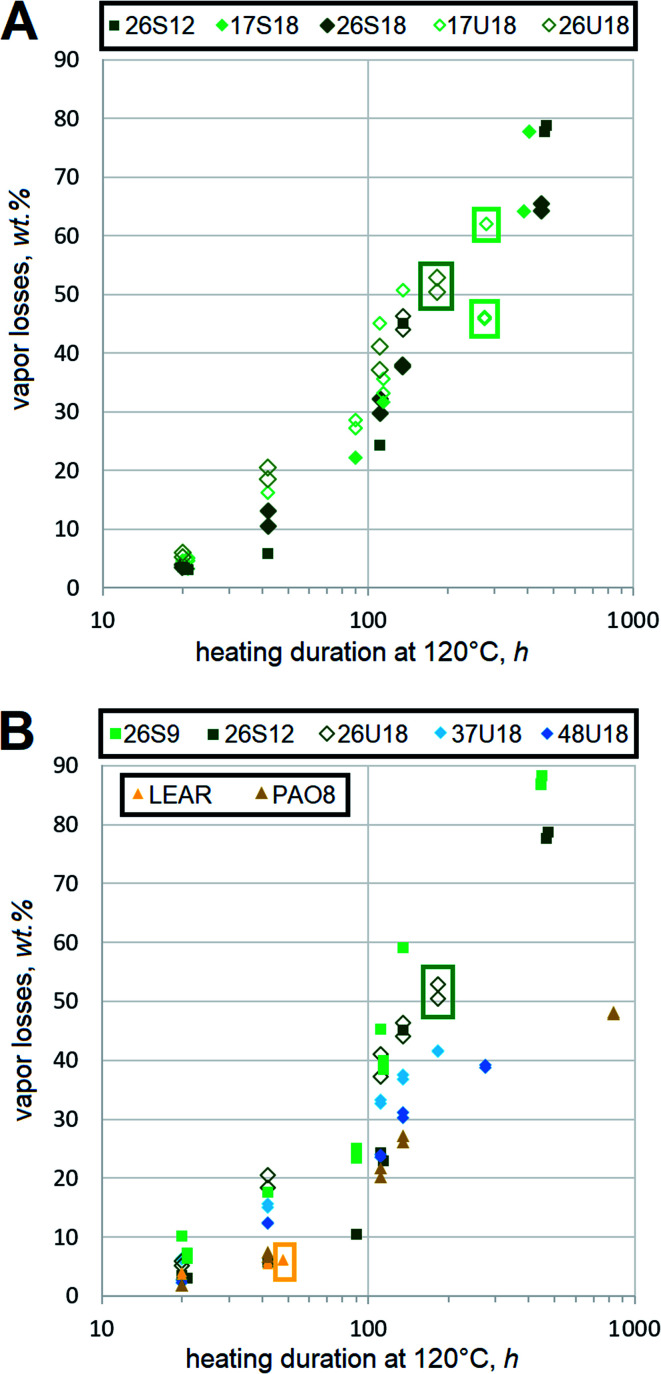
Vapor losses due to oxidative decomposition of (A) xxS18 and xxU18 dibasic esters of similar mol. wt and (B) other dibasic esters and conventional basestocks at 120 °C. Framed data points indicate that the test is terminated due to formation of insoluble residue.

Oxidative stability of HF is important, but not as critical as in engine oils, gear oils or many other lubricants. Frequently, heavy duty equipment can use vegetable oil-based HF for much longer durations than mineral oil HF despite dramatically poorer results of the former in standard bench oxidation tests.^34^ Antioxidant-fortified LEAR is a frequent choice for heavy duty HF, because operating temperatures of hydraulic systems are lower as a result of better heat capacity in esters than hydrocarbons.^35^ Therefore, this report mostly focuses on the qualitative ranking of oxidation trends of dibasic esters by comparing them to the conventional basestocks. One problematic outcome of HF degradation is oxidative decomposition, which is measured using a thin film procedure in this study. In order to minimize the effects of thermodynamic evaporation, saturated and monounsaturated dibasic esters of very similar mol. wt are compared side-by-side, Fig. 6(A). Although saturated esters 17S18 and 26S18 show lower decomposition rates, the difference from 17U18 and 26U18 is not dramatic. For example, after 100 h at 120 °C the vapor losses of xxU18 reach ∼40%, while those of xxS18 approach 30%.

Samples are tested in duplicates and all recorded values are displayed without averaging. Contribution of monounsaturation to the decomposition rates is still quite evident, but it does not appear that this could be extremely detrimental. Some difference between 2EH and 1-methylheptyl moieties can be identified. Dibasic esters with the latter seem to yield lower vapor losses, which agrees with the expectation that secondary ester linkages in 17S18 and 17U18 resist decomposition better than the primary ones in 26S18 and 26U18 respectively. However, more testing would be needed to prove this statistically.

Despite expectations that monounsaturation should accelerate oxidative scission with respect to aliphatic chains,^36^ the decomposition rates can still be compared to other basestocks. However, vapor losses of mineral basestocks should not be considered as oxidative decomposition under this protocol, because NOACK tests suggest that RL208 and 350SN might have relatively high amounts of lower mol. wt fractions. Therefore, only other dibasic esters as well as synthetic and vegetable basestocks are outlined. PAO and LEAR appears to produce volatiles somewhat slower, Fig. 6(B), but the difference from 17S18 is not dramatic. Dibasic esters 26S9 and 26S12, whose mol. wt is lower, show higher vapor losses. Due to the differences in mol. wt these rates should not be quantitatively attributed to the oxidative decomposition. However, functionally it can be assumed that amounts of volatiles, emitted from FAME-derived dibasic esters, are similar to those of synthetic basestocks.

Another outcome of HF degradation is the formation of insoluble residues. Thin film experiments show that all saturated dibasic esters remain liquid until the end of testing, even despite major vapor losses. For example, the vapor losses of 26S9 amount to more than 85% after 450 h, but the remaining film is still fully liquid. Mineral oils, PAO8 and squalane also show similar trends, Fig. 6(B). In contrast, monounsaturated esters tend to eventually form insoluble residues. Most xxU18 samples produce insolubles within 300 to 350 h of heating at 120 °C. Nevertheless, this is many times better than LEAR, which forms the residues within about 40 h under identical conditions. These observations support earlier conclusions of a similar study on monounsaturated diesters of 2EH and monounsaturated dicarboxylic α,ω-C22 acids,^27^ which found their stability fully sufficient for HF. Advantages of monounsaturated dibasic esters against LEAR would be even more evident in thinner films, where not only oxidative, but electrochemical aspects become important.^37^ Bearing in mind that LEAR is often used as a basestock for heavy duty HF, residue formation trends of monounsaturated dibasic esters are not worrisome. In general, their resistance to oxidative degradation should be fully sufficient to assure excellent stability.

### Other lubricant properties

A number of other technical aspects have to be considered when evaluating a specific dibasic ester as a candidate for HF basestock. Lubricant blenders expect to see some data on lubricity, corrosion resistance, water rejection, elastomer compatibility, biobased contents, biodegradation and maybe other aspects. The latter three strongly depend on the alcohol moiety, but if biobased 2EH alcohol is utilized, they should not be overly problematic. Lubricity, corrosion inhibition, oxidative degradation and water rejection are very dependent on basestock uniformity and functional additives. Unfortunately, these properties might be affected strongly negatively by various foreign components in the formulation. Therefore, optimization of additive types and concentrations should only be carried out when the selection of basestock is finalized. Therefore, manufacture-related parameters should be well established, such as amounts of byproducts (partial esters, unreacted alcohols, *etc.*) or contaminants (decomposition products, catalysts, *etc.*).

Dibasic esters are quite familiar to lubricant blenders and most of them have good understanding of which additives might be appropriate. Monounsaturated dibasic esters might respond somewhat differently than saturated ones, but additives designed for vegetable oils often address the issues with double bonds quite well. Bio-derived lubricity additives can also be used.^38^ In fact, a monounsaturated dibasic ester has already been formulated with antioxidants, anti-wear agents, corrosion inhibitors, passivators and other additives.^39^ The formulation performed successfully in comparison to brand name commercial HF. Although its basestock was synthesized using metathesis of high-oleic FAME, 2-butyl octanol was needed for esterification in order to achieve VG32.

Many pathways have been reported to produce 2-butyl octanol from bio-derived resources. It was obtained at 77% yield from 1-hexanol^40^ and also from 1-hexene.^41^ Both 1-hexanol^42,43^ and 1-hexene^44^ can be bio-derived. If 48U18 is fortified with polymeric additives for VI and pour point enhancement (a common practice in HF)^45^ the formulation should meet VG46 specifications as well.

Another approach to obtain 100% bio-derived HF basestocks could utilize a commercial 2EH from renewable resources and larger dibasic acid moieties. As discussed above, Fig. 3 implies that 2EH dibasic esters with α,ω-C22 or α,ω-C26 acids should have excellent viscosities for VG32 or VG46 respectively. It is worth noting that pure α,ω-C22 or α,ω-C26 acids do not have to be used. Esterification with 2EH can be also performed on the dibasic acid mixture, such as that obtained from metathesis products of mixed methyl oleate and erucate. The one with higher ratio of erucate should result in 100% bio-derived VG46 (after self-metathesis and transesterification with renewable 2EH), while that higher in oleate would eventually generate VG32.

Mixed alcohols can also be used for esterification. Previous results^38^ of VG32 formulations with of α,ω-C18:1, which was esterified with 1 : 1 blend of 2EH and 2-butyloctanol show good additive compatibility and imply great opportunities for high performance HF, utilizing monounsaturated dibasic esters. Furthermore, the benefits of monounsaturation are evident not only in major improvements of VI and low temperature fluidity. Tribological benefits of monounsaturated compounds are already recognized for their contribution to tribofilm formation.^46^ Therefore, monounsaturated basestocks become very appealing to HF blenders.

The feedstock for manufacturing such basestocks can be FAME, produced at existing biodiesel plants from a variety of oils, including industrial crops, and fractionated using conventional distillation. Among non-food oilseeds, crambe and camelina are especially promising, because they can be cultivated on low-value land^47^ and provide high amounts of erucic and gondoic acids, respectively. Valorization of fractionated FAME would add more value for biodiesel plant products, open up more markets for biobased materials and produce further socio-economic benefits due to excellent performance of monounsaturated dibasic esters in heavy duty lubricants.

## 5. Conclusions

A novel category of dibasic esters, based on monounsaturated linear α–ω dicarboxylic acids, was synthesized from FAME using metathesis during the initial stage. Viscosity grades for mainstream hydraulic fluids were targeted, mostly employing 2EH alcohol for subsequent transesterification. Viscometric measurements showed that the metathesis of methyl oleate (*i.e.* FAME with Δ9 double bond) led to VG22 (after transesterification with 2EH), while the data correlation suggested that such conversion of FAME with Δ11 unsaturation (*e.g.* methyl gondoate C20:1-Δ11) should lead to VG32 and FAME with Δ13 unsaturation (*e.g.* methyl erucate) to VG46 basestocks. Metathesis and transesterification of methyl oleate into VG32 was carried out by employing heavier alcohols than 2EH, just industrially that might require higher processing temperatures and deeper vacuum.

A series of basestock-related properties was tested and compared to widely used mineral oils, rapeseed oil and synthetic hydrocarbons. Resistance to heat thinning and VI was much higher, especially in monounsaturated esters, compared to conventional basestocks. Methyl branching improved low temperature fluidity somewhat, while ethyl and longer branches were dramatically more effective, as were double bonds. Monounsaturation was not excessively detrimental to oxidative decomposition and the dibasic esters were many times more resistant to oxidative solidification than LEAR. Their volatility was much lower than that of mineral oils and appeared comparable to synthetic basestocks. Consequently, their formulations can exceed high performance requirements, featured by petrochemical HF, while being able to approach the bio-derived contents of vegetable oil HF, which are not considered heavy duty due to rapid oxidation. High-erucic FAME might be particularly suitable as a feedstock for such monounsaturated dibasic esters and industrial considerations should be made for their commoditization.

## Conflicts of interest

There are no conflicts to declare.

## Supplementary Material
